# Changes in pore structure of coal caused by coal-to-gas bioconversion

**DOI:** 10.1038/s41598-017-04110-z

**Published:** 2017-06-19

**Authors:** Rui Zhang, Shimin Liu, Jitendra Bahadur, Derek Elsworth, Yi Wang, Guanglong Hu, Yanna Liang

**Affiliations:** 1 Department of Energy and Mineral Engineering, G3 Center and Energy Institute, The Pennsylvania State University, University Park, Old Main, PA 16802 USA; 2 Solid State Physics Division, Bhabha Atomic Research Centre, Mumbai, 400094 India; 3 Department of Civil and Environmental Engineering, 1230 Lincoln Drive, Southern Illinois University, Carbondale, IL 62901 USA

## Abstract

Microbial enhanced coalbed methane (ME-CBM) recovery is critically examined as a viable technology for natural gas recovery from coalbed methane (CBM) reservoirs. Since the majority of gas-in-place (GIP) is stored as an adsorbed phase in fine pores of coal matrix, the nano-pore structure directly influences gas storage and transport properties. Only limited studies have quantified the alteration of the nano-pore structure due to ME-CBM treatment. This study examines the evolution of the pore structure using a combination of small angle X-ray scattering (SAXS), low-pressure N_2_ and CO_2_ adsorption (LPGA) and high-pressure methane adsorption methods. The results show that the surface fractal dimension decreases for the two bioconverted coals compared to the untreated coal. After bio-treatment, the mesopore surface area and pore volume decrease with the average pore diameter increases, while the micropore surface area increases with pore volume decreases. Both inaccessible meso-/micropore size distributions decrease after bioconversion, while the accessible micropore size distribution increases, making a portion of closed micropore network accessible. In addition, the methane adsorption capacities increase after bio-treatment, which is confirmed by the increase of micropore surface area. A conceptual physical model of methanogenesis is proposed based on the evolution of the pore structure.

## Introduction

Biogenic gas is an important natural gas resource in coalbed methane (CBM) formations^1, 2^. Microbial enhanced coalbed methane (ME-CBM) recovery is one viable technology for enhancing gas recovery (EGR)^3–13^. Enhancing CBM production is important as production usually shows an extended flat tail in late depletion. This is principally controlled by gas desorption from nano-scale pores, gas diffusion through both nano- and micron-scale pores, and by secondary biogenic methanogenesis in cleats and fractures^14^. Specifically, for a particular ME-CBM field, the gas production potential can be influenced by the adsorption capacity of coal and coal maceral compositions, the availability of suitable biogeochemical organic matter for bacteria, favorable environmental conditions for microbial growth and evolution and the accessibility of a methanogenic microbial community to organic matter^15, 16^. CBM reservoirs are usually saturated with methane, and the pressure reduction in late-stage methane production follows the sorption isotherm when secondary biogenic methane is significant^6, 17, 18^.

Notably, several factors control the bioconversion of coal. These include coal surface area, coal chemical compositions, bioconversion rate, microbial habitability environment, and others. The methane formation rate is proportional to the cleat surface area accessible to microbes^19^. Different aromatic organic compounds of coal may have different methanogenesis pathways^20^. In anthracites, the mass of volatile matter correlates positively with the rate of methane production – this could be a controlling factor in ME-CBM operation^21^. However, higher methane release is found in low-volatile bituminous coals relative to high-volatiles coals – suggesting a rank dependence^22^. Additionally, vitrinite-rich and high-sulfur bituminous coals generate higher methane contents compared to vitrinite-poor and low-sulfur coals, both of which have high volatile contents^23^. In addition, the rate of coal bioconversion is conditioned by: the biomass of the methanogenic microbial community; the availability of suitable nutrients, salinity and pH of the formation water; favorable temperatures for bacteria to survive and thrive underground; the biogeochemical characteristics of the organic matter; and the accessibility and connectivity of pores and fractures^24–26^. The biogenic methane production rate increases with an increase in temperature and a decrease in pH^27^. A larger coal mass, smaller particle size and the availability of proper surfactants all result in higher methane biogeneration rates^9^. Similarly, the dewatering of CBM reservoirs may also stimulate the bioconversion process from coal to methane due to the oxidation of organic matter increasing the bioavailability^28^. Conversely, coal oxidation decreases total methane production which may not be a controlling factor for ME-CBM operations^29^.

Permeability of coal is one of the most important controlling factors in methane production, typically increasing during ME-CBM recovery and enabling effective transport and fresh conversion^19, 30^. Thus, shallow coals with high permeability are believed to be suitable for bioconversion^15^. However, the permeability of coal has a decreasing trend with an increase in bioconversion time for one *in situ* briquette molded packed-powder core flooding study^31^. Scanning electron microscopy (SEM) has been used to qualitatively investigate the type and shape of the methanogenic microbes at the coal surface^31–33^. Although the meso- and micropores are too small for bacteria to pass^19, 34^, the nutrient solution may be transported into these fine pores and affect the methane adsorption capacity of the coal matrix. The change in the micro-scale pore structure and its impact on methane storage and transport in the coal matrix during microbial stimulation, especially during physical treatments such as by nutrient-hydraulic fracturing in depleting CBM reservoirs, has not been studied.

Several techniques have been successfully used to investigate the pore structure of coal. SEM and transmission electron microscopy (TEM) have been used to investigate both pore size and shape over a limited window in scale^35^. Mercury intrusion porosimetry (MIP) together with low pressure N_2_ and CO_2_ gas adsorption (LPGA) have been used to quantitatively estimate pore volume, surface area, pore size distribution and fractal dimension of the macro-, meso- and micropores in the coal matrix^36^. X-ray computed tomography (X-ray CT)^37^ and nuclear magnetic resonance (NMR)^38^ imaging may also be applied to quantitatively evaluate the pore structure. In addition, small angle neutron scattering (SANS)^39^ as well as small angle X-ray scattering (SAXS)^40^ have been applied to image both the open and closed pore structure of coal. In this current study, changes in the meso- and micropore structures of coal matrix due to different bio-treatments were quantitatively characterized by combined SAXS and LPGA techniques. The methane adsorption capacities of both pre- and post-bio-treated samples were estimated by the volumetric sorption method. The objective of this study is to characterize the coal pore structure evolutions under different bio-treatment conditions. We will try to quantify the effect of the microbial-nutrient solution associated with bioconversion effect on pore structure changes. Such changes have potentially important impacts on the total methane production potential of ME-CBM reservoirs.

## Results

### Methane production from coal

**Figure 1:**
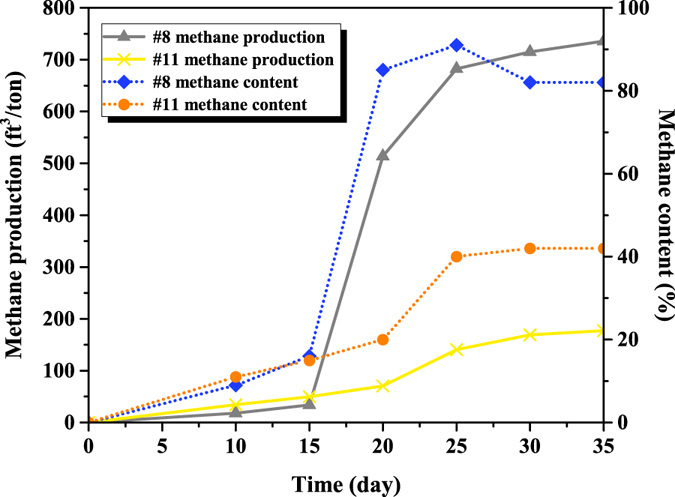
Methane content and production for two bioconverted coals.

The site where the coal blocks were collected was described previously^4^. In short, the coal with a heat content of 12,058 BTU/lb belongs to high volatile bituminous B rank. After the microbial community was added to the coal, it took 10 days until a significant content of methane could be detected in the headspace of the two microcosms (Fig. 1). After 15 days, however, methane content increased dramatically, especially for the #8 reactor. For this reactor, methane content peaked at 91% on day 25, decreased to 82% on day 30 and remained at that level for later days. Regarding the #11 reactor, methane content kept increasing until day 30. But the largest increase took place during day 20 and 25. As a result of bioconversion, cumulative methane production increased with time during the 35-day experimental period. The final methane release was 735.8 and 177.0 ft^3^/ton for #8 and #11 reactor, respectively. Therefore, temperature did have a significant effect on methane yield from coal.

### Characterization of chemical components

Changes in elemental composition of the coal samples due to bioconversion were recovered from assays (Table 1). The two treated samples had lower moisture content compared to untreated coal, with the treated #11 sample having higher volatile, carbon and oxygen contents and lower ash content compared to other two samples (control and treated #8). The fixed carbon, sulfur, hydrogen and nitrogen contents were near identical for all three samples. Based on the X-ray diffraction (XRD) results (Table 2), jarosite, melanterite and gypsum components were removed by biotreatment – interpreted as removal by dissolution in the added nutrient solution. The two treated coals had higher illite and pyrite contents and lower kaolinite content when compared with the untreated sample. Unexpectedly, the treated #11 sample had only trace quantities of calcite compared to the other two coals. This was possibly attributed to the reaction between calcite and the acid generated by anaerobic oxidation of ethanol^41^. In addition, it was observed that the amount of pyrite in the treated samples was greater than that in the untreated sample. Since Fe^3+^ serves as an electron acceptor in the bioconversion process, no methane could be produced until the supply of such electron acceptors was exhausted, signaling that Fe^3+^ was converted into Fe^2+^ ^42, 43^. This geochemical transformation may be the root cause for the increase in pyrite within the sample after biotreatment.

**Table 1:** Ultimate analysis for untreated and bioconverted coals.

Sample name	Moisture (%)	Volatile (%)	Fixed carbon (%)	Ash (%)	Sulfur (%)	Carbon (%)	Hydrogen (%)	Nitrogen (%)	Oxygen (%)
Untreated	3.62	37.03	50.77	8.58	3.52	66.19	4.49	1.39	12.21
Treated #8	2.63	38.68	50.40	8.29	3.32	68.11	4.64	1.66	11.35
Treated #11	2.98	42.44	51.70	2.88	3.21	69.40	4.58	1.60	15.35

**Table 2:** XRD result for untreated and bioconverted coals.

Sample name	Quartz (%)	Calcite (%)	Kaolinite (%)	Illite (%)	Pyrite (%)	Jarosite (%)	Melanterite (%)	Gypsum (%)	Total (%)
Untreated	22.5	7.9	25.8	7.8	4.3	20.2	8.1	3.4	100
Treated #8^a^	23.9	9.9	12.9	11.4	12.1	/	/	/	70.2
Treated #11^a^	29.2	0.3	13.2	18.2	10.7	/	/	/	71.6

^a^The percentage of mineral matters for two treated coals were normalized by carbon content and the disappearance of jarosite, melanterite and gypsum contents.

### Experimental and modeled scattering intensities

**Figure 2:**
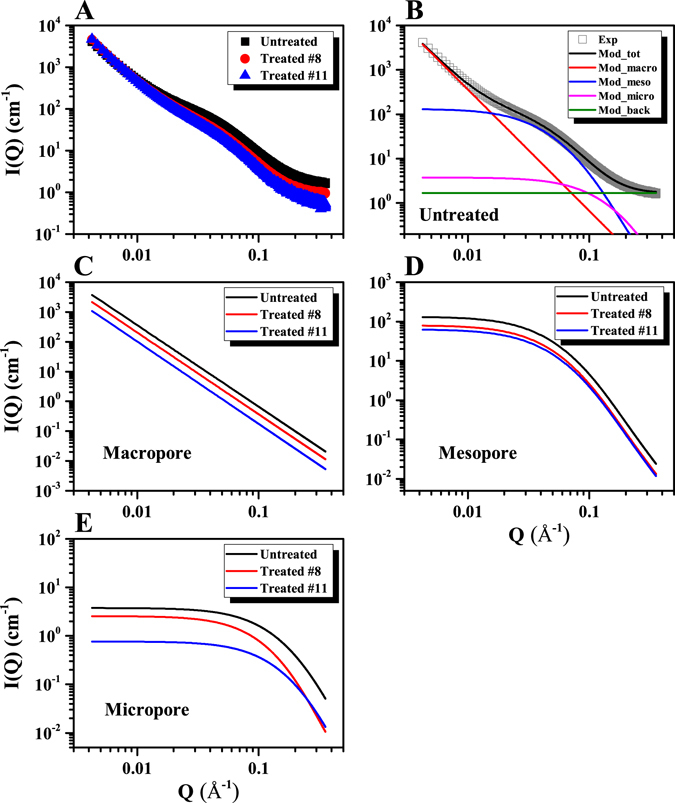
Scattering intensities for untreated and bioconverted coals. (**A**) Experimental data for three samples; (**B**) Representative modeling data for untreated coal; Scattering intensities of (**C**) macropore, (**D**) mesopore and (**E**) micropore for untreated and bioconverted coals (Exp: experimental data; Mod_tot: modeled total scattering intensity; Mod_macro: modeled scattering intensity for macropore; Mod_meso: modeled scattering intensity for mesopore; Mod_micro: modeled scattering intensity for micropore; Mod_back: background scattering intensity; Note: The macropore scattering intensity of the treated #8 and #11 samples was divided by 2 and by 4, respectively, for inter-comparison).

The scattering intensities, *I*(*Q*), are shown for the three coal samples in Fig. 2A as a function of the scattering vector *Q*. It is important to note that the real space distance *d* correlates with the reciprocal space distance *Q*, as *d* = 2*π*/*Q* ^44^. Specifically, for a fractal rock system, this real and reciprocal space correlation has an empirical relationship as *R* = 2.5/*Q* based on numerical simulation^45^. Thus, *Q* in the range 0.004 to 0.355 Å^−1^ corresponds to a range in pore radii between 0.7 and 58.8 nm. The *I*(*Q*) of all three coals are tightly distributed and nearly overlap in the low *Q* range (*Q* ≤ 0.01 Å^−1^) as shown in Fig. 2A. It is notable that *I*(*Q*) in the low *Q* region is dominated by scattering from macropores. This suggests that the alteration of the macropore morphology is minimal for both of the treated (#8 and #11) coal samples. However, the *I*(*Q*) of both bio-converted coals decreases in comparison to the untreated coal in the medium and high *Q* ranges (*Q* > 0.01 Å^−1^). It is noted that the medium and high *Q* scattering contribution comes from the meso- and micropores, respectively. This indicates that the morphologies of the meso- and micropores are altered during the bioconversion process.

The measured *I*(*Q*) can be modeled by combining the three scattering contributions, *I*(*Q*)*s*, from the macropores (subscript *ma* for the low *Q* region), from the mesopores (*me* for the medium *Q* region) and from the micropores (*mi* for the high *Q* region). Thus, the total *I*(*Q*) can be expressed as:

1 $$I(Q)={I}_{{\rm{ma}}}(Q)+{I}_{{\rm{me}}}(Q)+{I}_{{\rm{mi}}}(Q)+{I}_{{\rm{b}}}$$

where *I*_m*x*_(*Q*) are the various scattering intensities and *I*_b_ is the constant background arising due to chemical inhomegeities at a very small length scale (few Å). Generally, the coal matrix is a fractal system at a larger length scale with significant complexity and heterogeneity. A power law scattering model, usually used to describe the fractal nature of porous media^46^, can be used to represent the macropore scattering *I*_ma_(*Q*) in the small *Q* region as:

2 $${I}_{{\rm{ma}}}(Q)={C}_{{\rm{p}}}{Q}^{-\alpha }$$

where *C*_p_ is the *Q*-independent constant and depends on both the specific surface area of the pore-matrix interface and the scattering contrast between pores and rock matrix; *α* is the power law exponent that describes the fractal nature of the porous system. However, in the large *Q* region, the scattering intensities due to meso- and micropores, *i.e. I*_me_(*Q*) and *I*_mi_(*Q*) can be represented by polydisperse spherical pore (PDSP) scattering models. The scattering intensity for the PDSP model can be expressed as^44^:

3 $${I}_{{\rm{PDSP}}}(Q)=N{({\rm{\Delta }}{\rho }^{\ast })}^{2}{\int }^{}{V}^{2}(r)D(r)P(Q,r)dr$$

where *N* is the number density of pores; *V*(*r*) is the volume of spheres with a radius *r*; (Δ*ρ* ^*^)^2^ is the scattering contrast between pores and the surrounding rock matrix which can be expressed as:

4 $${({\rm{\Delta }}{\rho }^{\ast })}^{2}={({\rho }_{{\rm{s}}}^{\ast }-{\rho }_{{\rm{p}}}^{\ast })}^{2}={({\rho }_{{\rm{s}}}^{\ast })}^{2}({\rho }_{{\rm{p}}}^{\ast }\cong 0)$$

Here ${r}_{{\rm{p}}}^{\ast }$ is the scattering length density (SLD) of the pore which can be assumed to be zero for empty pores; ${\rho }_{{\rm{p}}}^{\ast }$ is the SLD of the solid matrix. The SLD of material for X-ray scattering can be estimated as:

5 $${\rho }^{\ast }={I}_{{\rm{e}}}{\rho }_{{\rm{e}}}=\frac{{{\rm{N}}}_{{\rm{A}}}d}{M}{N}_{{\rm{e}}}{I}_{{\rm{e}}}$$

where *I*_e_ is the scattering amplitude of a single electron; *ρ*_e_ is the electron density; N_A_ is Avogadro’s constant; *d* is the bulk density; *M* is the molecular weight; *N*_e_ is the number of electrons. It is noted that the chemical composition and bulk density of the organic matter are assumed as C_137_H_97_O_9_NS and 1.2 g/cm^3^, respectively for the SLD estimation of the Illinois coal matrix. *D*(*r*) is the lognormal size distribution which can be expressed as:

6 $$D(r)=\frac{1}{\sqrt{2\pi {\sigma }^{2}{r}^{2}}}\exp (-\frac{\mathrm{ln}\,{(\frac{r}{{r}_{0}})}^{2}}{2{\sigma }^{2}})$$

where *σ* is the polydispersity index and *r*_0_ is the median pore radius. Finally, *P*(*Q*, *r*) is the form factor for the sphere and can be written as:

7 $$P(Q,r)=9{[\frac{\sin (Qr)-Qr\cos (Qr)}{{(Qr)}^{3}}]}^{2}$$

Eq. 1 was used to fit the experimental SAXS data for both untreated and treated coals. A nonlinear regression model is applied for the fitting of the data. For the untreated coal sample, the modeled SAXS results, together with the measured data, are shown in Fig. 2B. The modeled SAXS profile agrees well with the measured data for the entire range of *Q* for the untreated coal sample. The scattering contributions from macro-, meso-, and micro-pores, as well as the background to the total scattering intensity are also depicted in Fig. 2B. It is noted that the background to the experimental data that was used in the SAXS modeling was based on the scattering intensity at the highest *Q* for each sample. This was 1.69 cm^−1^ for the untreated coal, 0.99 cm^−1^ for the treated #8 coal and 0.53 cm^−1^ for the treated #11 coal. For all three samples, the modeled scattering contributions for each type of pores, *i.e*. macro-, meso- and micro-pores, are shown in Fig. 2C,D and E. It is evident from Fig. 2C that power law scattering due to the macropores shows a linear profile in a log-log plot for each coal, as expected from Eq. 2. It is clear from Fig. 2D that the scattering intensity from the mesopores *I*_me_(*Q*) decreases without significant change in the functionality of the scattering profile for the bio-converted samples. This indicates that the mesopore volume fraction decreases without appreciable change in pore size and shape during the bioconversion of the coals. Conversely, apparent from Fig. 2E is that the scattering profiles of the micropores undergo significant changes during bioconversion. Unlike the mesopore scattering profiles, the functionality of the scattering profiles of the micropores is altered significantly as a result of bio-treatment. This identifies that not only the pore volume fraction, but also the size of the micropores, are altered significantly for the treated coal samples. These changes in the pore morphology does not depend on the microbial activity directly as size of the microbes is quite large compared to the size of mesopores and micropores. However, meso-/micropore morphology is altered indirectly during the bioconversion process which is related to the coal type and rank, nutrient solution, temperature and pH^22, 24–26^.

### Low-pressure N_2_ and CO_2_ adsorption isotherms

**Figure 3:**
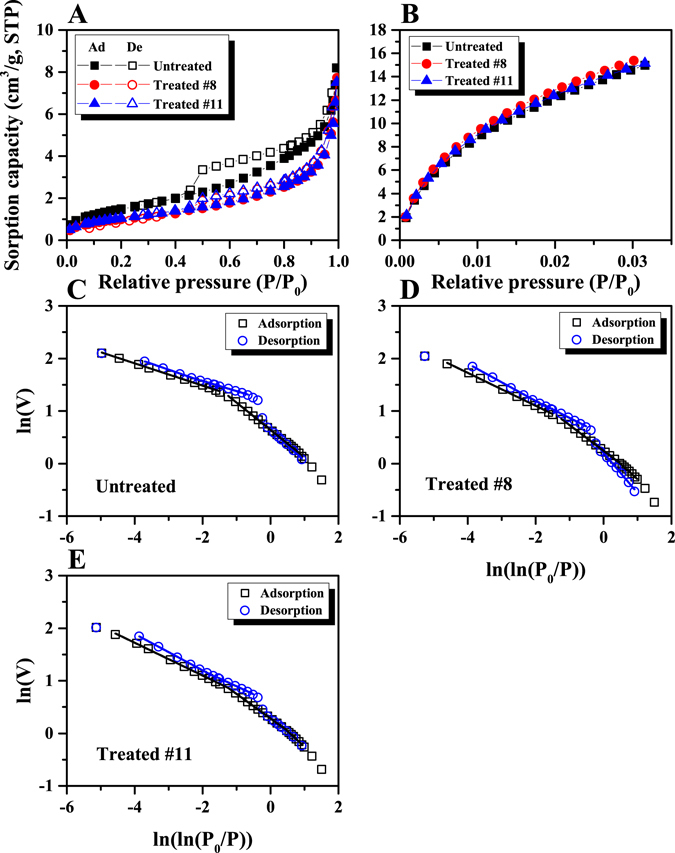
LPGA isotherms for untreated and bioconverted coals. (**A**) N_2_ sorption isotherms; (**B**) CO_2_ sorption isotherms (Ad: adsorption; De: desorption); FHH fractal analysis from low-pressure N_2_ sorption data for untreated and bioconverted coals. (**C**) Untreated coal; (**D**) Treated #8 coal; (**E**) Treated #11 coal.

Figure 3 shows the low-pressure N_2_ and CO_2_ sorption isotherms for both untreated and bio-converted coals. Both N_2_ and CO_2_ adsorption capacities increased with the increase of relative pressure for all three coals (Fig. 3A and B). At low relative-pressure, CO_2_ sorption capacities of the two bio-converted coals are slightly greater than that of untreated coal as shown in Fig. 3B (relative pressure range, *P*/*P*_0_ < 0.032). However, the N_2_ sorption isotherms of the bio-treated coals overlap, where the N_2_ sorption capacities are smaller than that of untreated coal sample over the entire relative pressure range (Fig. 3A). Based on our experimental results, these findings were interpreted as the bioconversion only influencing pores in the mesopore size range or above while the micropore structure remains unaffected – based on the low-pressure data. This observation contradicts observations from the SAXS data, where there is a significant change in the micropore volume and size for the bio-converted samples. Since SAXS can detect both open and closed pores while LPGA can only detect fluid-accessible pores, this discrepancy could result from the bioconversion treatment only affecting inaccessible micropores and leaving the interconnected pores unaffected – an unusual outcome. Detailed discussion of this enigmatic response is given in the following Section.

### Surface fractal dimension

Pore structure of coal is both heterogeneous and anisotropic with fractal characteristics. Fractal pore structure can be quantitatively described by its fractal dimension. In general, the surface fractal dimension *D*_s_ varies from 2 to 3 with the volumetric fractal dimension *D*_m_ smaller than or equal to 3. Additionally, the pore space fractal dimension *D*_p_ represents the spatial irregularity of a given pore network. In this study, the Frenkel-Halsey-Hill (FHH) fractal model was used to estimate *D*_s_ from the low-pressure N_2_ adsorption-desorption data. The FHH equation can be expressed as^47^:

8 $$V\propto {[\mathrm{ln}(\frac{{P}_{0}}{P})]}^{-s}$$

where *V* is the sorption capacity; *P* is the equilibrium pressure; *P*_0_ is the saturated vapor pressure; and *s* is a constant which represents the fractal nature of the object. There are two methods to estimate *D*_s_ based on the FHH equation, which are^48–50^:

9 $$V\propto {[\mathrm{ln}(\frac{{P}_{0}}{P})]}^{\frac{{D}_{{\rm{s}}}-3}{3}}$$

10 $$V\propto {[\mathrm{ln}(\frac{{P}_{0}}{P})]}^{{D}_{{\rm{s}}}-3}$$

It is noted that the Eq. 9 can be used to estimate *D*_s_ when adsorption occurs to depths of the order of a single monolayer to several multilayers. In this case, the adsorption is dominated by Van der Waals forces. However, when capillary condensation becomes prevalent and pore-filling adsorption becomes the dominant mechanism then Eq. 10 can be used to estimate *D*_s_^49^. In this study, *D*_s_ was estimated by linearly regressing the relationship between In*V* and In[In(*P*_0_/*P*)] using Eq. 10. In this, 2 to 3 linear sections were used to estimate *D*_*s*_ based on N_2_ adsorption and desorption isotherms for these three coals shown in Fig. 3C,D and E. Low-pressure sorption data fit well for all linear sections for these tested samples where R^2^ is greater than 0.995. The fractal results are shown in Table 3. It was found that *D*_s1_ (*D*_s_ in the linear section at small relative pressure), *D*_s2_ (at medium relative pressure) and *D*_s3_ (at large relative pressure) for the treated coals are all smaller than those for the untreated sample except the *D*_s3_ of the treated #11 coal. This suggests that either the surface of the meso-/macropores became smooth, or closed pores disappeared, which may result from consumption of either the bacteria or the nutrient solution. It is notable that since the fractal dimension estimated by SAXS is representative of the mass/volume, rather than the surface, the results were not shown in this study to compare with low-pressure sorption data.

**Table 3:** Fractal parameters for untreated and bioconverted coals.

Sample	N_2_ adsorption			N_2_ desorption		
	*D*_s1_	*D*_s2_	*D*_s3_	*D*_s1_	*D*_s2_	*D*_s3_
Untreated	2.48	/	2.79	2.40	2.80	2.78
Treated #8	2.41	2.51	2.69	2.24	2.67	2.64
Treated #11	2.41	2.53	2.69	2.45	2.70	2.65

### Pore volume and surface area

The meso- and micro-pore volumes and surface areas of the coal can be estimated based on low-pressure N_2_ and CO_2_ isotherms^47, 51^. In this study, the Brunauer-Emmett-Teller (BET) equation was used to estimate mesopore surface area and the Barrett-Joyner-Halenda (BJH) equation was used to estimate mesopore volume based on N_2_ adsorption data for all tested coals. Additionally, the Dubinin-Radushkevich (D-R) equation was used to estimate surface area of the micropores and the Dubinin-Astakhov (D-A) equation was used to estimate micropore volume based on CO_2_ adsorption data. The estimated pore volume and surface area results for untreated and bio-converted coals are shown in Table 4. It was found that both BET surface area and BJH mesopore volume decreased from 5.51 to ~3.79 m^2^/g and from 0.0126 to ~0.0117 m^3^/g, which is consistent with the decrease of surface fractal dimension for the treated coals (Table 3). Conversely, the average mesopore width increased from 8.94 to ~12.35 nm after bioconversion treatment. For micropores, no obvious change was observed for both D-R surface area and D-A pore volume as a result of bio-treatment. The D-R surface area increased slightly from 125.06 to ~127.98 m^2^/g while the D-A pore volume decreased slightly from 0.0600 to ~0.0586 m^3^/g. These findings suggest that the bioconversion mainly affects the accessible mesopores rather than the micropores, based on the LPGA data.

**Table 4:** Pore structure parameters for untreated and bioconverted coals.

Sample	N_2_ adsorption			CO_2_ adsorption	
	BET surface area (m^2^/g)	BJH mesopore volume (cm^3^/g)	BJH average pore width (nm)	D-R micropore surface area (m^2^/g)	D-A micropore volume (cm^3^/g)
Untreated	5.51	0.0126	8.94	125.06	0.0600
Treated #8	3.70	0.0119	12.75	129.54	0.0598
Treated #11	3.88	0.0115	11.96	126.42	0.0575

### Pore size distribution

**Figure 4:**
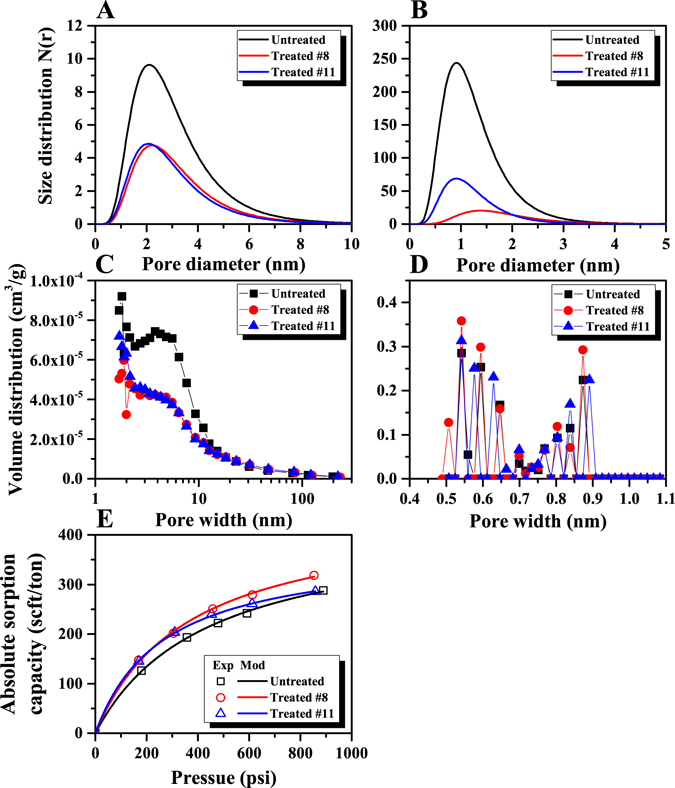
Meso- and micro-pore size distributions from SAXS data for untreated and bioconverted coals. (**A**) Mesopore; (**B**) Micropore. Meso- and micro-pore size distributions from LPGA data for untreated and bioconverted coals. (**C**) Mesopore; (**D**) Micropore. (**E**) Methane absolute adsorption isotherms and Langmuir-modeled results for untreated and bioconverted coals (Exp: experimental data; Mod: modeled results).

The meso- and micropore size distributions were evaluated by both SAXS and LPGA for both untreated and bio-treated coals. The estimated meso-/micro-pore size distributions based on the SAXS scattering profiles are shown in Fig. 4A and B. The pore number density, N(r), obtained from the SAXS measurements decreases from 141.54 cm^−3^ (untreated) to 72.43 cm^−3^ (treated #8) and 70.56 cm^−3^ (treated #11) for the mesopores. Similarly, the number density of micropores decreases from 1410.85 cm^−3^ (untreated) to 161.94 cm^−3^ (treated #8) and 383.55 cm^−3^ (treated #11) for the micropores. This suggests that the number densities of both micro- and meso-pores decrease after bio-treatment. In addition, the mean pore radius shows a very small change for both the meso- and micro-pore size distribution from before to after bio-treatment. These findings suggest that the bioconversion affects both meso- and micro-pores.

Based on the LPGA data, the BJH mesopore volume distribution estimated by N_2_ sorption decreased after biogenic methanogenesis when pore width is smaller than 20 nm (Fig. 4C) – this is consistent with mesopore size distribution obtained from SAXS data. However, the micropore volume distribution obtained from the CO_2_ sorption data shows a small increase after bioconversion (Fig. 4D), which is consistent with the D-R micropore surface area whereas it is inconsistent with the results obtained from the SAXS. This discrepancy in pore size distribution in the micropore range between SAXS and low-pressure CO_2_ adsorption measurements is due to the fact that SAXS can detect both open and closed pores while low-pressure sorption techniques can only detect open pores^39^. Since, in bituminous coals, most of pores in the micropore range could be closed^39, 52^, the comparison of the micropore size distributions obtained by SAXS and LPGA indicates that bioconversion mainly affects the closed micropores for the tested coals. Based on the results of the mesopore size distribution, the consistency between SAXS and N_2_ sorption data suggests that a major fraction of the mesopores are interconnected. Thus, the shrinkage of both the closed micropore and open mesopore structure may be caused by either nutrient penetration or the removal of microbes.

### Methane adsorption capacity

Figure 4E shows the absolute adsorption capacity and modeled results for untreated and bio-converted coals. It is noted that only the excess adsorption capacity can be directly estimated by the volumetric sorption method (see detailed methodology of excess sorption capacity estimation from previous studies^53^) However, absolute adsorption capacity is usually used to evaluate total gas-in-place (GIP) for the estimation of methane production from CBM reservoirs. To estimate absolute sorption capacity from the detectable excess sorption capacity, the density ratio between free gas and adsorbed gas can be used to correct. Thus, the absolute adsorption capacity *V*_ab_ can be estimated as:

11 $${V}_{{\rm{ab}}}=(1-\frac{{\rho }_{{\rm{b}}}}{{\rho }_{{\rm{a}}}})/{V}_{{\rm{ex}}}$$

where *V*_ex_ is the excess adsorption capacity; *ρ*_b_ and *ρ*_a_ are the bulk and adsorbed phase densities, respectively. It is noted that bulk gas density was estimated from an equation of state (EOS) and adsorbed gas density was assumed constant at 0.421 g/cm^3^ for the correction for methane^53^. The absolute adsorption capacity of the two bio-treated coals are greater than for the untreated sample over the entire pressure range. This is caused by the increase of micropore surface area^54^, where there are more sorption sites for treated coals compared to untreated coal. Specifically, the methane sorption capacity of the treated #8 sample is almost identical to that for the treated #11 sample when the equilibrium pressure is smaller than ~300 psi. While the methane sorption capacity of the treated #8 sample is greater than that for the treated #11 sample when pressure is above ~400 psi.

The absolute sorption capacity of the three coal samples is represented by a Langmuir isothermal, expressed as^55^:

12 $${V}_{{\rm{ab}}}=\frac{{V}_{{\rm{L}}}P}{{P}_{{\rm{L}}}+P}$$

where *P* is the equilibrium pressure, *V*_L_ is the Langmuir volume; and *P*_L_ is the Langmuir pressure. The Langmuir model fits well for the sorption capacities of all these coals as shown in Fig. 3E, where the modeled parameters are reported in Table 5. It was found that the Langmuir volume *V*_L_ of the treated #8 coal is greater than that for the untreated coal, while *V*_L_ of the treated #11 coal is smaller than that for the untreated sample. The Langmuir pressure *P*_L_ of the two treated coals are both smaller than that for the untreated sample. The changes of V_L_ and P_L_ suggest that the bio-treatment alters the profile of the adsorption isotherms. Based on the Fig. 3E, the sorption capacity increase for the treated coals at the tested pressure range, which is helpful to elevate gas production at low depletion wellbore pressure.

**Table 5:** Langmuir parameters for untreated and bioconverted coals.

Sample	*V*_L_ (ft^3^/ton)	*P*_L_ (psi)	R^2^
Untreated	425.21	433.59	1
Treated #8	448.94	360.87	0.999
Treated #11	374.60	264.06	1

## Discussion

### A conceptual pore structure evolution mechanism with the bio-treatment

**Figure 5:**
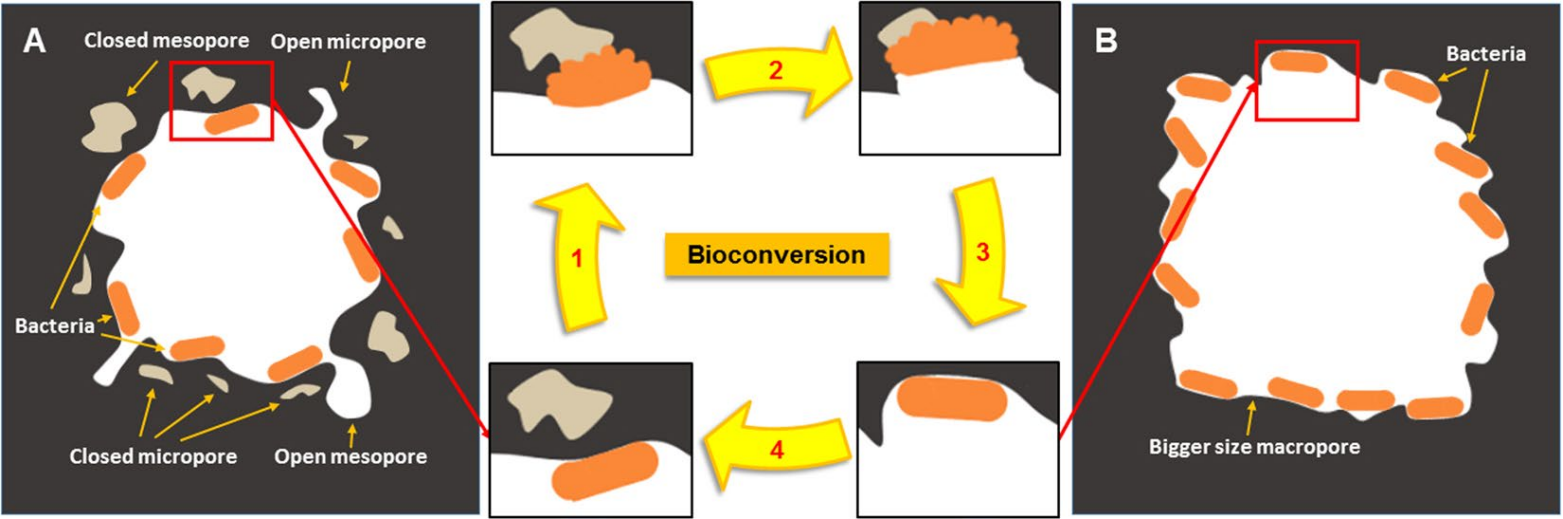
A conceptual mechanism for bioconversion of coal. (**A**) Pore structure before biogenic treatment. There are several isolated and accessible meso-/micropores surrounding the macropore surface where bacteria cannot go into. (**B**) Pore structure after biogenic treatment. Both isolated and accessible meso-/micropores vanished (Fig. 4A,B,C and D) and macropore surface becomes smoother (Table 3). (Note: The cyclic flow chat in the middle shows the process of coal-to-gas bioconversion: 1) Isolated meso-/micropres became accessible; 2) Bacteria continuously consumed the coal matrix near the surface of meso-/micropores; 3) Newly generated accessible meso-/micropres vanished; 4) Another bioconversion cycle began).

As previously mentioned, the discrepancy in the sense of the change in pore volumes/structure between SAXS and LPGA may be attributed to changes in the size and connectivity of the micro-scale inaccessible pores. We propose a conceptual model for pore structure evolution resulting from the bio-treatment, as shown in Fig. 5. This model considers that the bacteria are initially confined to the macropores which are connected to accessible meso-/micropores (Fig. 5A). A portion of the closed micro-/mesopores are close to the internal surfaces of the macropores prior to bioconversion (Fig. 5A). After the bio-treatment, both closed and open micro-/mesopores disappear due to the progress of bio-consumption and the size of the macropore is correspondingly enlarged (Fig. 5B). This hypothesized bio-induced pore evolution model is based on a comparison of the pore structure both before and then after methanogenesis and in particular of in the meso-/micropore size range. The results of the fractal analysis show that the *D*_s_ of the treated coals are smaller than that for untreated coal, suggesting roughness as well as self-similarity of the pore surface slightly decreases for the bio-treated coals (Table 3). This finding can be interpreted as either closed pores becoming connected to the network or disappearing, and/or that contact with bacteria and/or the nutrient solution smoothed the pore surface. A second line of evidence is from the results of the pore volume and surface area analyses (Table 4). Mesopore surface area and volume, and micropore volume all decreased while the average mesopore width increased, suggesting a reduction of the accessible micro-/meso-pore volume. The results of the micro-/meso-pore size distributions, based on SAXS and LPGA, show that both closed and accessible mesopores decreased during the biogenesis (Fig. 4A and C). However, the volume of closed micropores significant decreased (Fig. 4B) and accessible micropores showed a slight increase (Fig. 4D) in volume during biogenic conversion. The observed increase in accessible micropores is consistent with the observed increase in methane adsorption capacity following bio-treatment (Fig. 4E), where most of the gas is adsorbed in the micropores. These findings also indicate that only the surface layer of the macropore is accessible to the bacteria and that the bio-treatment will consume a portion of the solid coal. This consumption could possibly render the closed micro-/meso-pores accessible to gas or cause them to vanish depending on the intensity of the effect, as illustrated in Fig. 5. Certainly, the size of the macropore will be enlarged due to the consumption of the solid coal. However, further studies will be needed to confirm this hypothesis for the alteration of pore structure.

## Summary

Alteration of the pore structure for both untreated and for two different samples of bio-treated coals was characterized by a combination of small angle X-ray scattering (SAXS), low-pressure N_2_ and CO_2_ adsorption and high-pressure methane adsorption methods. The surface fractal dimension *D*_s_ for the mesopores was estimated by both SAXS and low-pressure N_2_ adsorption. The pore volume and surface area of the micro-/mesopores were estimated by low-pressure N_2_ and CO_2_ adsorption. The micro-/meso-pore size distributions were estimated by both SAXS and low-pressure N_2_ and CO_2_ adsorption. Methane adsorption capacity was estimated by the volumetric method. Based on these results, several conclusions are summarized as below:

1. *D*_s_ decreases for each equivalent pore size and in each treated sample compared to that for the untreated sample.
2. Both BET surface area and BJH pore volume decrease while average pore width increases for treated coals. For the micropore structure, the D-R micropore surface area increases while the D-A micropore volume decreases for these two treated samples.
3. The number density of both inaccessible meso-/micropores decreases after bio-treatment, but the number density of accessible micropores increases after the bio-treatment making a portion of inaccessible pores newly accessible.
4. Methane sorption isotherms increase over the experimental pressure range which is consistent with the increase in micropores.
5. A conceptual mechanism of the effects of bio-treatment is proposed that is consistent with the observed evolution of pore structure.

## Materials and Methods

### Sample preparation

Fresh coal blocks were collected from an Illinois coal mine and pulverized to powder with particle sizes less than 74 μm. A fraction of the coal powder was kept as untreated control samples for baseline comparison. The remaining portion of the powdered sample was used for a biotreatment study that was published already^56^. Briefly, the bioconversion study was conducted to identify optimal value for temperature, coal particle size, ethanol concentration and coal loading. For this purpose, a total of 29 reactors were established under different conditions. The temperature was either 24, 28 or 32 °C since the *in situ* temperature varies between 24 and 30 °C. Coal particle size was either less than 37, 55.5 or 74 µm. Concentration of ethanol was 100, 200 or 300 mM. Coal loading was 200, 300 or 400 g/L. It needs to be noted that these four parameters and the range tested for each were based upon results from a screening study^56^. The screening study demonstrated that out of 12 parameters, these four were critical ones affecting methane yield from coal. To each microcosm under different conditions, an inoculum initially developed from the *in situ* formation water was added. This community has a diverse bacterial population and much less diverse archaeal species^4^. A nutrient solution based on a MS medium recipe^57^ but simplified^56^ was added to each microcosm. Once the microcosms (100-mL serum bottles) were set up, the headspace gas in each bottle was monitored at different time intervals following our previously reported procedures^4, 56, 58, 59^. For this study, samples from the two microcosms, #8 and #11 were used in order to characterize the change in pore structure following treatment at different temperatures. The #8 and #11 reactors were maintained at 32 °C and 28 °C, respectively. Both the #8 and #11 reactors contained coals with a particle size of <55.5 µm and received 100 mM of ethanol. Ethanol, compared to other organic solvents and possibly electron donors, had statistically significant positive effect on methane release from coal according to our previous studies^56^ and other reports^60^. Its exact role in coal bioconversion is still being investigated.

### Small angle X-ray scattering experiments

The SAXS experiments were conducted using a PANalytical Empyrean θ-θ diffractometer in the Materials Research Institute (MRI) at Penn State University. Powdered coal samples were used with a particle size of <55.5 μm to record scattering intensity *I*(*Q*) as a function of the scattering vector *Q* at room temperature (24 °C) and under vacuum (10^−3^ kPa). The X-ray beam was generated by a Cu Kα source with a wavelength of 1.54 Å. The scattering signal was collected by a PIXcel3D detector in 1D scanning mode. The effective range of *Q* varies between 0.004 and 0.355 Å^−1^. Additionally, *I*(*Q*) were corrected for empty sample holder scattering before data interpretation.

### Low-pressure N_2_ and CO_2_ adsorption experiments

The LPGA experiments were conducted using the ASAP 2020 Plus Physisorption technique in the MRI at Penn State University. Untreated and treated powdered coal samples with a particle size of <55.5 μm were measured by both low-pressure N_2_ adsorption-desorption at −196 °C and low-pressure CO_2_ adsorption at 0 °C to recover the meso- and micropore structure.

### High-pressure methane adsorption

The high-pressure methane adsorption experiments were conducted at the Energy Institute at Penn State University. Volumetric sorption was measured in combined stainless steel sample and reference cells with two Omega pressure transducers. In total, three powdered coal samples with particle sizes of <55.5 μm were measured for six pressure steps from zero pressure to ~900 psi at 35 °C.

### Headspace gas analyses

Headspace gas analyses were conducted in the same way as reported before^59^. Briefly, to maintain a 1 atm pressure in each reactor and release overpressure caused by microbial activities, a stainless steel needle was inserted to each microcosm headspace at different time points. The needle was connected to a 50-mL gas tight syringe. Gas volume in the syringe was recorded and used for calculation of methane yield. The molar contents of methane in the reactor headspace were analyzed through a 17A GC (Shimadzu, Columbia, MD, USA). This GC was equipped with a 60 m × 0.53 mm RT-Msieve 5A porous layer molecular sieve (Restek, Bellefonte, PA, USA) and a flame ionization detector with argon being the carrier gas with a flow rate of 10.1 mL/min. The isothermal zone temperatures for the injector and detector were set at 75 °C and 310 °C, respectively. The retention time for methane was 4.73 min. Calibration curves for methane (5–99%) was established using standard gases (Air Liquide, Plumsteadville, PA, USA).
